# ﻿Two new species of the genus *Paradexamine* (Crustacea, Amphipoda, Dexaminidae) from Korean Waters

**DOI:** 10.3897/zookeys.1117.85644

**Published:** 2022-08-11

**Authors:** Xin Zhang, Kyung-Won Kim, Young-Hyo Kim

**Affiliations:** 1 Department of Biological Sciences, Dankook University, Cheonan, 31116, Republic of Korea Dankook University Cheonan Republic of Korea

**Keywords:** Crustacea, *Paradexamineacuta* sp. nov., *Paradexaminerotundogena* sp. nov., taxonomy

## Abstract

Two dexaminid amphipod species belonging to the genus *Paradexamine* were collected from Korean waters. After observation and identification compared with related congeners, these two species are revealed to be new to science. In comparative identification, one of the new species, *P.acuta***sp. nov.** is similar to *P.houtete* in having an acutely rounded lobe and posteroventrally pointed coxa 7. However, this new species is distinguished from *P.houtete* in having a larger number of medial setae on the propodus of gnathopod 1 and a rounded basis of pereopod 6. This new species is also very similar to *P.marlie* s.l. Hirayama from Japanese waters, and *P.marlie* s.l. might be re-established or synonymized with *P.acuta***sp. nov.** in the future. The other new species, *P.rotundogena***sp. nov.** is similar to *P.tafunsaka* in having a rounded eye lobe; however, it is distinguished from *P.tafunsaka* in having an elongate carpus on gnathopod 2 and the differently shaped basis of pereopod 7. A key to the five Korean species of *Paradexamine*, including the two new species, is also provided.

## ﻿Introduction

Twelve genera of the family Dexaminidae have been reported worldwide (Lowry and Myers 2017; Horton et al. 2022). Among these genera, the genus *Paradexamine* Stebbing, 1899 is the largest genus of the family and is easily distinguished from other genera in having a distinct cephalic lobe and dorsal teeth on the pleonites. In *Paradexamine*, the number of dorsal pleon teeth increases with growth of juveniles, but the teeth remain fairly stable in their count in adults (Barnard 1972a). *Paradexamine* is divided into two groups based on characteristics of the cephalic lobe, which is either rounded or acute. Of the 46 paradexaminid species in the world, there are approximately three times as many species with the cephalic lobe rounded as those with the cephalic lobe acute.

Dexaminids occur in various habitats but are most common among algae, sand, gravel, or rubble habitats in relatively shallow water (Myers and LeCroy 2009). Paradexaminids mainly live in algae habitats and have phototactic (light preference) responses, so often appear in shallow Korean waters in night surveys using light traps.

Hitherto, three *Paradexamine* species with an acute cephalic lobe have been recorded from Korea: *P.fraudatrix* Tzvetkova, 1976, *P.gigas* Hirayama, 1984, and *P.jindoensis* Kim & Lee, 2008 (Kim et al. 2006; Kim and Lee 2008). In addition, we add two new species, *P.acuta* and *P.rotundogena* to the Korean dexaminid fauna. Furthermore, a paradexaminid with a rounded cephalic lobe is recorded for the first time in Korean waters. A key to the Korean *Paradexamine* species is also given.

## ﻿Materials and methods

**Figure 1.**
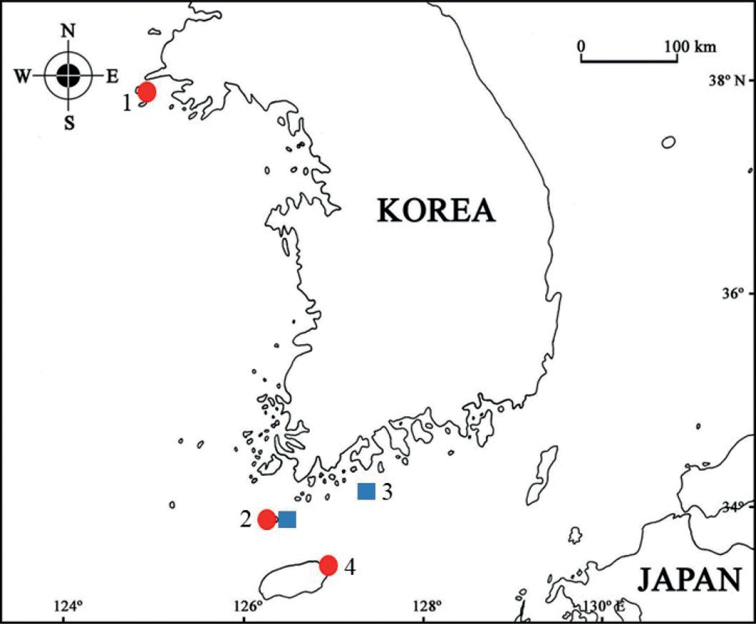
Distribution of the *Paradexamineacuta* sp. nov. (red circle) and *P.rotundogena* sp. nov. (blue square): 1 = Baengnyeongdo Island, 2 = Chujado Island, 3 = Geomundo Island, 4 = Jongdal Port, Jejudo Island.

Materials for this study were obtained by scuba diving from Baengnyeongdo, Chujado, Geomundo, and Jejudo Islands located off Korea’s west and south coasts from 2018–2022 (Fig. 1). The specimens were fixed with 95% ethanol and dissected in glycerol on Cobb’s aluminum hollow slides. Permanent mounts were made using polyvinyl lactophenol with lignin pink added. Pencil drawings were made and measurements were taken using a drawing tube mounted on an Olympus SZX 12 stereomicroscope and an Olympus BX 51 interference contrast compound microscope. Line drawings were produced using the program ‘Graphic’. Body length was measured from the tip of the rostrum to the posterior end of the urosome, along the dorsal parabolic line of the body. In this study, we followed the scheme of Barnard (1972a), who counted dorsal pleonal teeth from the rear to the front, commencing with pleonite 4 and progressing forward to pereonite 6. Number “3” means the segment has 1 mediodorsal and 2 dorsolateral teeth; “1” means the only mediodorsal tooth is present. Type specimens are deposited at the Marine Biodiversity Institute of Korea (MABIK) in Seocheon, Korea and the Department of Biological Science, Dankook University (DKU), Cheonan, Korea.

## ﻿Taxonomy

### ﻿Order Amphipoda Latreille, 1816

**Suborder Amphilochidea Boeck, 1871**

#### Family Dexaminidae Leach, 1814

##### ﻿Genus Paradexamine

Taxon classificationAnimaliaAmphipodaDexaminidae

Stebbing, 1899

6738A948-2C66-501E-836C-CBC5556667EF

###### Species composition.

*Paradexamineacuta* sp. nov.; *P.aequiserrata* (Myers & LeCroy, 2009); *P.alkoomie* (Barnard, 1972a); *P.barnardi* (Sheard, 1938); *P.bisetigera* (Hirayama, 1984); *P.churinga* (Barnard, 1972a); *P.dandaloo* (Barnard, 1972a); *P.echuca* (Barnard, 1972a); *P.excavata* (Ledoyer, 1984); *P.exilis* (Myers & LeCroy, 2009); *P.fissicauda* (Chevreux, 1906); *P.flindersi* (Stebbing, 1888); *P.fraudatrix* (Tzvetkova, 1976); *P.frinsdorfi* (Sheard, 1938); *P.gigas* (Hirayama, 1984); *P.goomai* (Barnard, 1972a); *P.houtete* (Barnard, 1972b); *P.indentata* (Ledoyer, 1978); *P.jindoensis* (Kim & Lee, 2008); *P.lanacoura* (Barnard, 1972a); *P.latifolia* (Ren, 2006); *P.levitelson* (Myers & LeCroy, 2009); *P.linga* (Barnard, 1972a); *P.marlie* (Barnard, 1972a); *P.massa* (Myers & LeCroy, 2009); *P.maunaloa* (Barnard, 1970); *P.micronesica* (Ledoyer, 1979); *P.miersi* (Haswell, 1885); *P.moorehousei* (Sheard, 1938); *P.mozambica* (Ledoyer, 1979); *P.muriwai* (Barnard, 1972b); *P.nana* (Stebbing, 1914); *P.narluke* (Barnard, 1972a); *P.orientalis* (Spandl, 1923); *P.otichi* (Barnard, 1972a); *P.pacifica* (Thomson, 1879); *P.quadratus* (Myers & LeCroy, 2009); *P.quarallia* (Barnard, 1972a); *P.rewa* (Myers, 1985); *P.rotundogena* sp. nov.; *P.ronngi* (Barnard, 1972a); *P.saxeta* (Myers & LeCroy, 2009); *P.serraticra* (Walker, 1904); *P.setigera* (Hirayama, 1984); *P.sexdentata* (Schellenberg, 1931); *P.tafunsaka* (Myers, 1995); *P.thadalee* (Barnard, 1972a); *P.windarra* (Barnard, 1972a).

##### ﻿ Paradexamine acuta sp. nov.

Taxon classificationAnimaliaAmphipodaDexaminidae

E362C138-C1E6-561E-BA2B-0E21E6C26071

https://zoobank.org/4FCC0F2F-0AFE-4200-94FF-F8418E61342B

**Figure 2.**
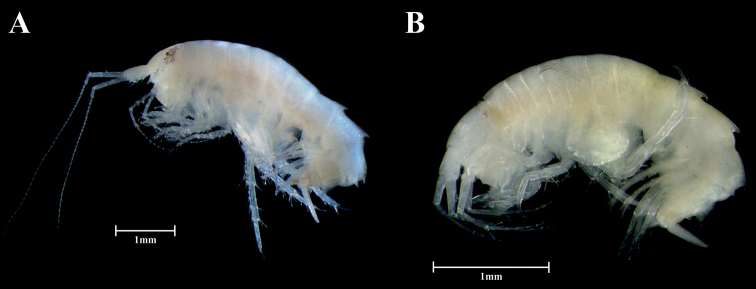
**A***Paradexamineacuta* sp. nov., adult female, habitus **B***Paradexaminerotundogena* sp. nov., adult female, habitus. Scale bar: 1.0 mm (**A, B**).

**Figure 3.**
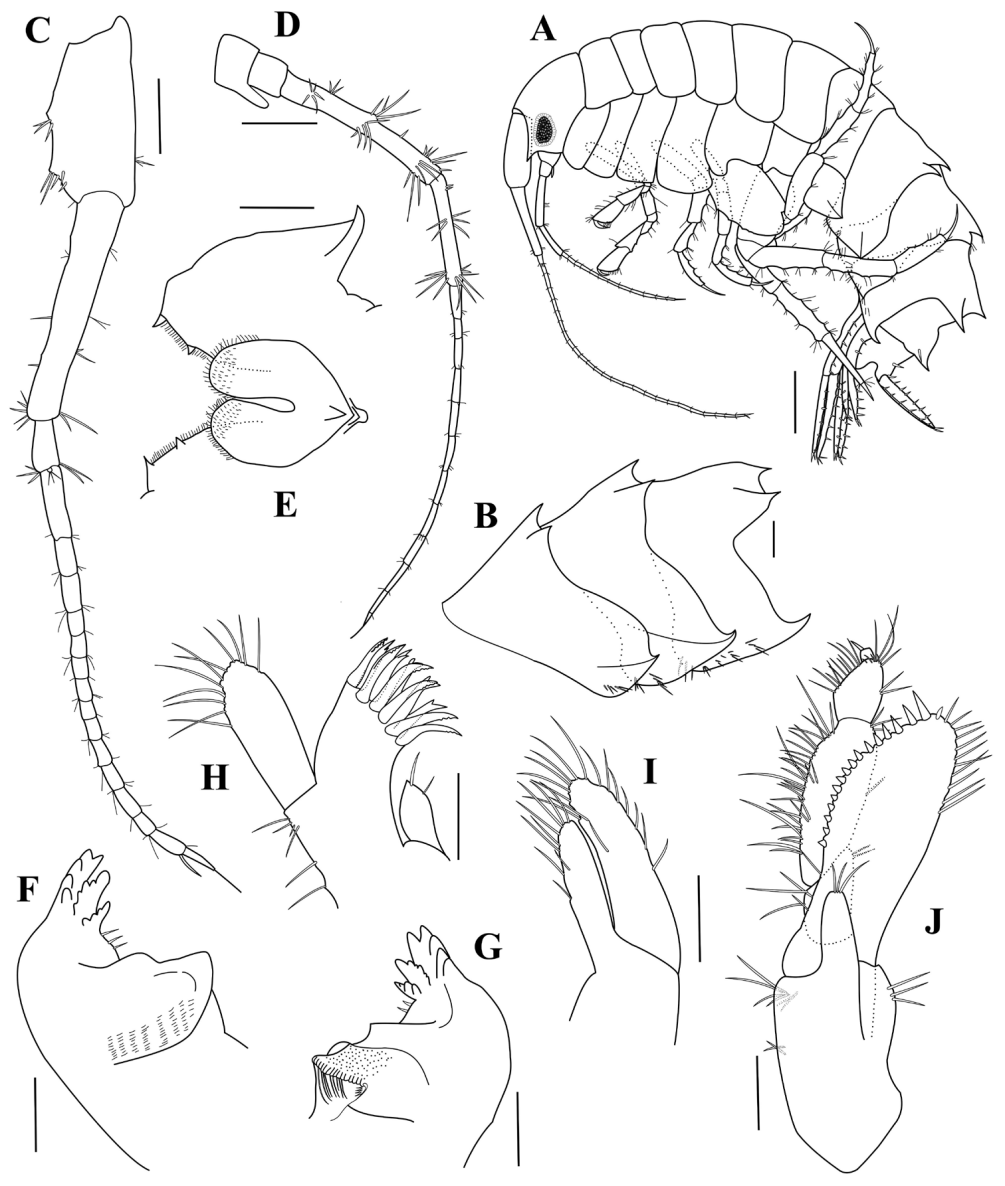
*Paradexamineacuta* sp. nov., holotype, adult female, MABIK CR00250813, 5.0 mm **A** habitus **B** pleonal epimera 1–3 **C** antenna 1 **D** antenna 2 **E** lower lip **F** left mandible **G** right mandible **H** maxilla 1 **I** maxilla 2 **J** maxilliped. Scale bars: 0.5 mm (**A**); 0.2 mm (**B**); 0.1 mm (**C–J**).

**Figure 4.**
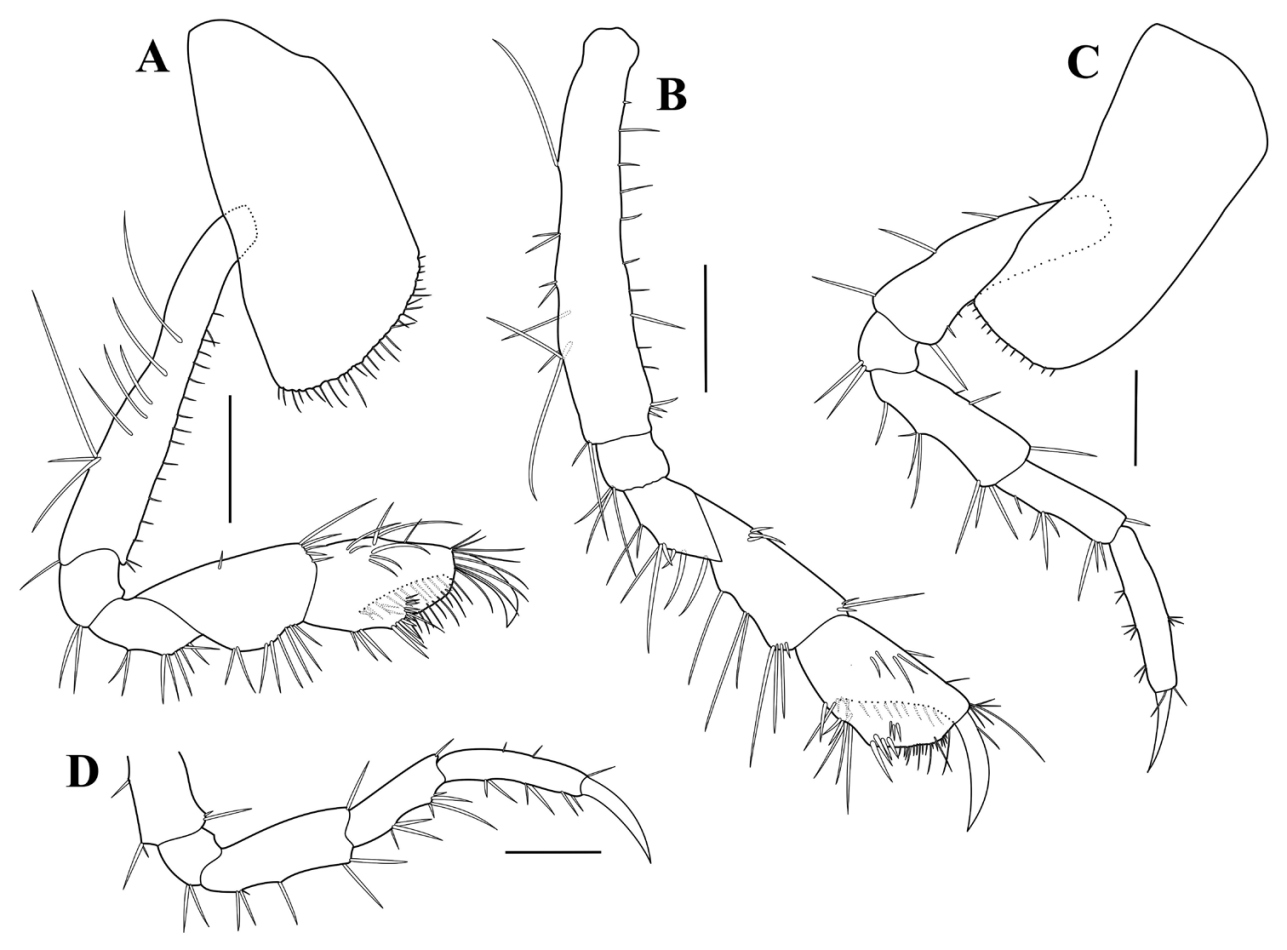
*Paradexamineacuta* sp. nov., holotype, adult female, MABIK CR00250813, 5.0 mm **A** gnathopod 1 **B** gnathopod 2 **C** pereopod 3 **D** pereopod 4. Scale bars: 0.2 mm (**A, B**), 0.1 mm (**C, D**).

**Figure 5.**
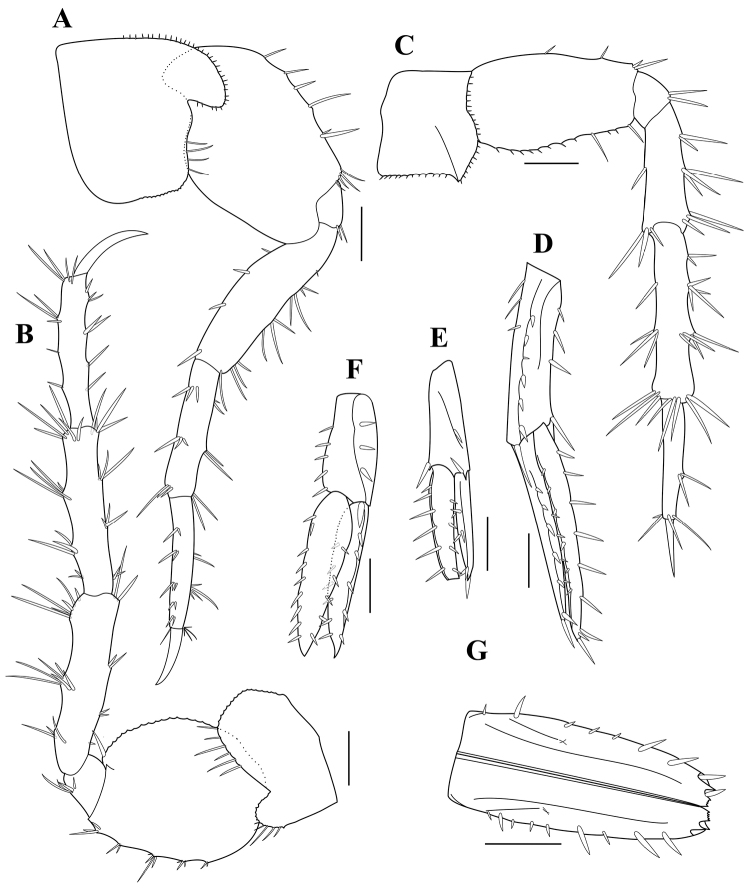
*Paradexamineacuta* sp. nov., holotype, adult female, MABIK CR00250813, 5.0 mm **A** pereopod 5 **B** pereopod 6 **C** pereopod 7 **D** uropod 1 **E** uropod 2 **F** uropod 3 **G** telson. Scale bar: 0.2 mm (**A–G**).

Figs 2A , 3 , 4 , 5 Korean name: Ppyo-jok-yeop-ga-si-but-eun-kko-ri-yeop-sae-u, new

###### Type material.

***Holotype***: female, 5.5 mm, MABIK CR00250813, Korea, Incheon, Baengnyeongdo Island, Dumujin, 37°58'36"N, 124°37'09"E, 13 August 2020, scuba collection in red alga *Gelidium* sp., depth 5–10 m, S.G. Lee & Y.H. Kim leg. ***Paratypes***: 3 females, 4.6 mm, 5.1 mm, and 5.4 mm, DKUAMP202201, same station data as holotype.

###### Additional material examined.

22 females, DKUAMP202202, Korea, Incheon, Baengnyeongdo Island, 37°58'26"N, 124°38'39"E, Y.H. Kim leg., 12 August 2020. 1 female, Korea, Chujado Island, 33°57'13"N, 126°18'08"E, Z. Xin, K.W. Kim, & Y.H. Kim leg., 27 August 2021. 7 females, Korea, Jejudo Island, Jongdal Port, 33°29'49"N, 126°54'41"E, Y.H. Kim leg., 5 February 2022.

###### Diagnosis.

Lateral cephalic lobe acute. Eye small. Dorsal pleonites tooth formulae 1-3-3-3-0, rear to front. Outer lobe of lower lip with two corns. Maxilla 1, inner plate with two setae. Maxilliped, inner plate lacking lateral setae. Antenna 1, peduncular article 2 1.25 times article 1. Gnathopod 1, propodus subovate, medial side with oblique row of 11 or 12 setae; palm oblique. Coxa 7, posterodistal corner acutely produced. Telson deeply cleft, with lateral and apical spines.

###### Description.

***Holotype*, female**, MABIK CR00250813. Body (Fig. 3A) length about 5.5 mm. Ocular lobe acutely produced. Eye small, subovate. Pereonites smooth.

Pleonites 1–3 (Fig. 3B) dorsal tooth formulae 1-3-3-3-0, rear to front; pleonal epimera 1‒3 each with posteroventral tooth, gradually enlarging distally; epimeron 1 with oblique row of 5 setae ventrally; epimeron 2 similar to pleonal epimeron 1 but with two clusters of setae anteroventrally; epimeron 3 excavate posteriorly, with five clusters of setae ventrally.

Antenna 1 (Fig. 3C) slightly longer than half as long as body length; peduncular articles rectangular, length ratio of peduncular articles 1‒3 = 1.00: 1.25: 0.25; accessory flagellum small, with three apical setules; flagellum subequal in length to peduncle, 15-articulate.

Antenna 2 (Fig. 3D) 0.78 times as long as antenna 1; gland corn well developed; peduncular articles 3‒5 setaceous facially, length ratio = 1.00: 5.00: 2.90; flagellum 1.13 times as long as peduncle, 12-articulate.

Lower lip (Fig. 3E), inner lobe subovate, coalescent proximally, rounded apically; outer lobe with two corns, mandibular process upturned and acute.

Left mandible (Fig. 3F), incisor produced forward, with five blunt teeth; lacinia mobilis bifid, upper part with five teeth, lower part with four teeth; three accessory spines placed between lacinia mobilis and molar process; molar process massive, developed.

Right mandible (Fig. 3G) similar to left mandible, except two accessory spines placed between lacinia mobilis and molar process.

Maxilla 1 (Figs 3H), inner plate with two simple setae apically; outer plate six denticulate and five bifid tooth-like spines apically; palp broad, with 11 simple setae.

Maxilla 2 (Fig. 3I), inner plate shorter and narrower than outer plate, with nine setae; outer plate with 14 setae overall.

Maxilliped (Fig. 3J), inner plate elongate, about one-third as long as outer plate, with three apical setae; outer plate elongate-ovate, slightly extending beyond end of palp article 3; inner margin crenulate, with 12 conical teeth which gradually increase in size toward distal end; distal half of outer margin with a row of 11 simple setae; palp 4 articulate, rather slender, inner margin setaceous, extending outer plate.

Gnathopod 1 (Fig. 4A), coxa trapezoidal, ventral margin rounded, with unequal setae; basis slender, about half as long as gnathopod 1, with nine simple setae posteriorly; ischium small, subrectangular, 0.75 times as long as merus; carpus 1.17 times as long as propodus; propodus subovate, with oblique row of 11 setae medially, palm oblique, with a row of short setae, delimited by a group of four spines; dactylus falcate, fitting palm.

Gnathopod 2 (Fig. 4B) similar to gnathopod 1, but longer and slenderer than gnathopod 1.

Pereopod 3 (Fig. 4C), coxa subrectangular, one-third as wide as long, ventral margin setose; length ratio of articles 2‒7 = 1.00: 0.18: 0.62: 0.50: 0.67: 0.35.

Pereopod 4 (Fig. 4D) similar to pereopod 3.

Pereopod 5 (Fig. 5A), coxa subquadrate, bilobate, anterior rounded lobe protruding downward, with short setae; basis with longish ovate form, posteroventral lobe rounded downward, reaching somewhat near distal margin of ischium, with several clusters of long to short spines along anterior margin; ischium to dactylus slender, setose; length ratio of articles 2‒7 = 1.00: 0.18: 0.90: 0.68: 0.66: 0.20.

Pereopod 6 (Fig. 5B), coxa 6 bilobate, similar to coxa 5, but shallower than coxa 5; basis ovate, posterior margin rounded and finely serrulate; length ratio of articles 2‒7 = 1.00: 0.20: 1.10: 1.02: 1.15: 0.59.

Pereopod 7 (Fig. 5C), coxa subquadrate, with acutely produced posteroventrally; basis subrectangular, narrow, width 0.57 times length; length ratio of articles 2‒7 = 1.00: 0.24: 0.71: 1.07: 0.71: 0.36.

Uropod 1 (Fig. 5D), peduncle subrectangular, subequal to outer ramus, with six dorsolateral, four medial, three basofacial, and one apicolateral large spines; inner ramus slightly longer than outer ramus.

Uropod 2 (Fig. 5E) 0.64 times as long as uropod 1; peduncle subequal to outer ramus, with two dorsolateral and one apicomedial spines; inner ramus slightly longer than outer ramus, apical portion broken.

Uropod 3 (Fig. 5F) longer and broader than uropod 2; peduncle 0.69 times as long as outer ramus, with three dorsolateral, five medial, and one apicolateral large spines; both rami subequal in length.

Telson (Fig. 5G) longish, 2.37 times as long as wide, thoroughly cleft, lateral margin with a row of unequal spines, apical margin truncate, with serrulation and one spine.

**Male.** Unknown.

**Immature female**, 3.0 mm, DKUAMP202202. Gnathopod 1, propodus subovate, with oblique row of eight setae medially; pereopod 6, basis ovate, posterior margin rounded and finely serrulate; coxa 7 pointed posteroventrally; telson, lateral margin with a row of seven spines.

###### Etymology.

The species name is derived from the Latin *acutus* (= sharp, pointed), referring to the acute cephalic lobe and posteroventral acute projection on coxa 7.

###### Remarks.

The new species *Paradexamineacuta* sp. nov. resembles *P.houtete* J.L. Barnard, 1972b from New Zealand, *P.jindoensis* Kim & Lee, 2008 from Jindo Island, Korea, *P.gigas* Hirayama, 1984, *P.marlie* s.l., and *P.micronesica* Ledoyer, 1978 from Tomioka Bay, Japan, in having acute ocular lobe and dorsal pleonites tooth formulae 1-3-3-3-0, rear to front (Table 1). However, this new species is distinguished from its congeners in the following characteristics (compared with the characteristics of congeners in parentheses): 1) inner plate of maxilla 1 with five lateral setae (vs without setae in *P.gigas*, *P.houtete*); 2) maxilliped, inner plate without lateral setae (vs seven lateral setae in *P.jindoensis*); 3) gnathopod 1 having medial setae on propodus with 10 or 11 setae (vs four or five setae in *P.gigas*, *P.houtete*, *P.micronesica*); 4) pereopod 6 with basis ovate, roundly produced posteriorly and with serrulations (vs tapering posterodistally in *P.gigas*, *P.houtete*, *P.micronesica*); 5) pereopod 7 with coxa pointed posteroventrally (vs rounded posteroventrally in *P.gigas*, *P.jindoensis*); 6) pereopod 7, basis subrectangular and narrow (vs subovate in *P.gigas*, elongate-ovate and moderate in *P.micronesica*).

**Table 1.** Morphological characters of *Paradexamineacuta* sp. nov. and related species.

Species Characters	*P.gigas* (♂)	*P.houtete* (♀)	*P.jindoensis* (♀)	*P.marlie* s.l. (♀)	*P.micronesica* (♂?)	*P.acuta* sp. nov. (♀)
Body length	3.5 mm	3.7 mm	5.5 mm	3.5 mm	3.0 mm	5.5 mm
Pleonites tooth formulae	1-3-3-3-0	1-3-3-3-0	1-3-3-3-0	1-3-3-3-0	1-3-3-3-0	1-3-3-3-0
					1-3-3-1	
Ocular lobe	acute	acute	acute	acute	acute	acute
Lower lip, corn	1 cone	2 cones	2 unequal cones	2 cones	1 cone	2 cones
Mandibular process	blunt, weakly developed	apically hooked	apically hooked	apically hooked	blunt, weakly developed	apically hooked
Mandible, accessory spines	no spine	2 spines	2 or 3 tiny spines	?	no spine	2 or 3 spines
Maxilla 1, palp	5 apical setae	8 setae	6 apical setae	7 apical setae	5 apical setae	11 apical setae
Maxilla 2, inner margin of inner plate	no seta	no seta	5 setae	4 setae	4 setae	5 setae
Maxilliped, inner plate	no lateral seta	no lateral seta	7 lateral setae	no lateral seta	no lateral seta	no lateral seta
Maxilliped, outer plate	11 conical teeth	12 conical teeth	16 conical teeth	12 conical teeth	11 conical teeth	17 conical teeth
Antenna 1, articles 1 & 2 ratio	1.0 : 1.5	1.0 : 1.1	1.0 : 1.5	1.0 : 1.1	1.0 : 2.0	1.0 : 1.3
Gnathopod 1, interior setae of propodus	4 setae	5 setae	8 setae	9 setae	4 setae	10 or 11 setae
Gnathopod 1, carpus & propodus ratio	1.0 : 0.9	1.0 : 0.9	1.0 : 0.9	1.0 : 1.3	1.0 : 1.0	1.0 : 0.9
Gnathopod 2, carpus & propodus ratio	1.0 : 0.7	1.0 : 0.8	1.0 : 0.9	1.0 : 0.8	1.0 : 0.5	1.0 : 0.9
Pereopod 6, basis	tapering posterodistally	excavated posterodistally	roundly produced posteriorly	roundly produced posteriorly	slightly tapering posterodistally	roundly produced posteriorly
Coxa 7	rounded posteroventrally	pointed posteroventrally	rounded posteroventrally	pointed posteroventrally	pointed posteroventrally	pointed posteroventrally
Pereopod 7, basis	subovate	subrectangular	subrectangular, narrow	subrectangular, narrow	elongate-ovate, moderate	subrectangular, narrow
Telson	cleft, roundish marginally, 1 lateral spine	slender and longish, 4 or 5 lateral spines	slender and longish, 14 or 15 lateral spines	elongate-ovate, 7 lateral spines	slender and longish, 2 lateral spines	slender and longish, 7 or 9 lateral spines
Distribution	Tomioka bay, Japan	New Zealand	Jindo Island, Korea	Tomioka bay, Japan	Tomioka bay, Japan	Korea
References	Hirayama, 1984	Barnard, 1972b	Kim & Lee, 2008	Hirayama, 1984	Hirayama, 1984	Present study

In general, *Paradexamineacuta* sp. nov. is very similar to *P.marlie* s.l. from Japanese waters as described and figured by Hirayama (1984). *Paradexaminemarlie* s.l. from Japan differs from the original description of *P.marlie* from Australia (Barnard 1972a) in having the dorsal pleonites tooth formulae 1-3-3-3-0 and coxa 7 posteroventrally pointed. In many ways, including these two major characteristics, *P.marlie* s.l. from Japan is more similar to *P.acuta* sp. nov. However, as we have not had the opportunity to examine Hirayama’s (1984) type material, we cannot confidently determine if they are the same species. Nevertheless, *P.marlie* s.l. Hirayama could be re-established or synonymized with *P.acuta* sp. nov. in the future.

###### Distribution.

South Korea (Baengnyeongdo Island, Chujado Island, Jejudo Island)

##### ﻿ Paradexamine rotundogena sp. nov.

Taxon classificationAnimaliaAmphipodaDexaminidae

2DC44A49-D2CA-5DDE-9991-32D6731AD407

https://zoobank.org/BE364F87-4E67-4023-9F95-B094D345F835

**Figure 6.**
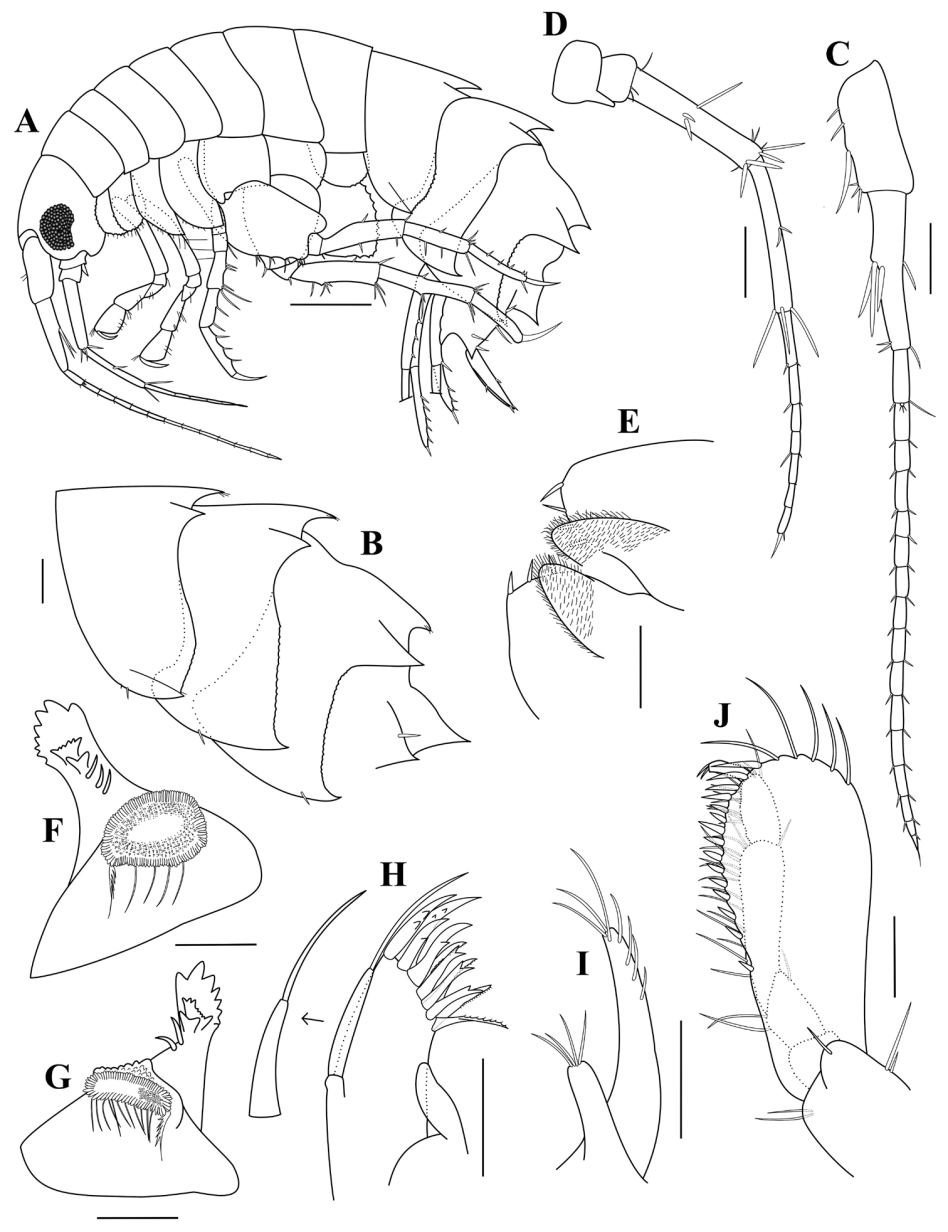
*Paradexaminerotundogena* sp. nov., holotype, adult female, MABIK CR00250814, 5.5 mm **A** habitus **B** pleonal epimera 1–3 **C** antenna 1 **D** antenna 2 **E** lower lip **F** left mandible **G** right mandible **H** maxilla 1 **I** maxilla 2 **J** maxilliped. Scale bars: 0.5 mm (**A**); 0.2 mm (**B–D**); 0.1 mm (**E–J**).

**Figure 7.**
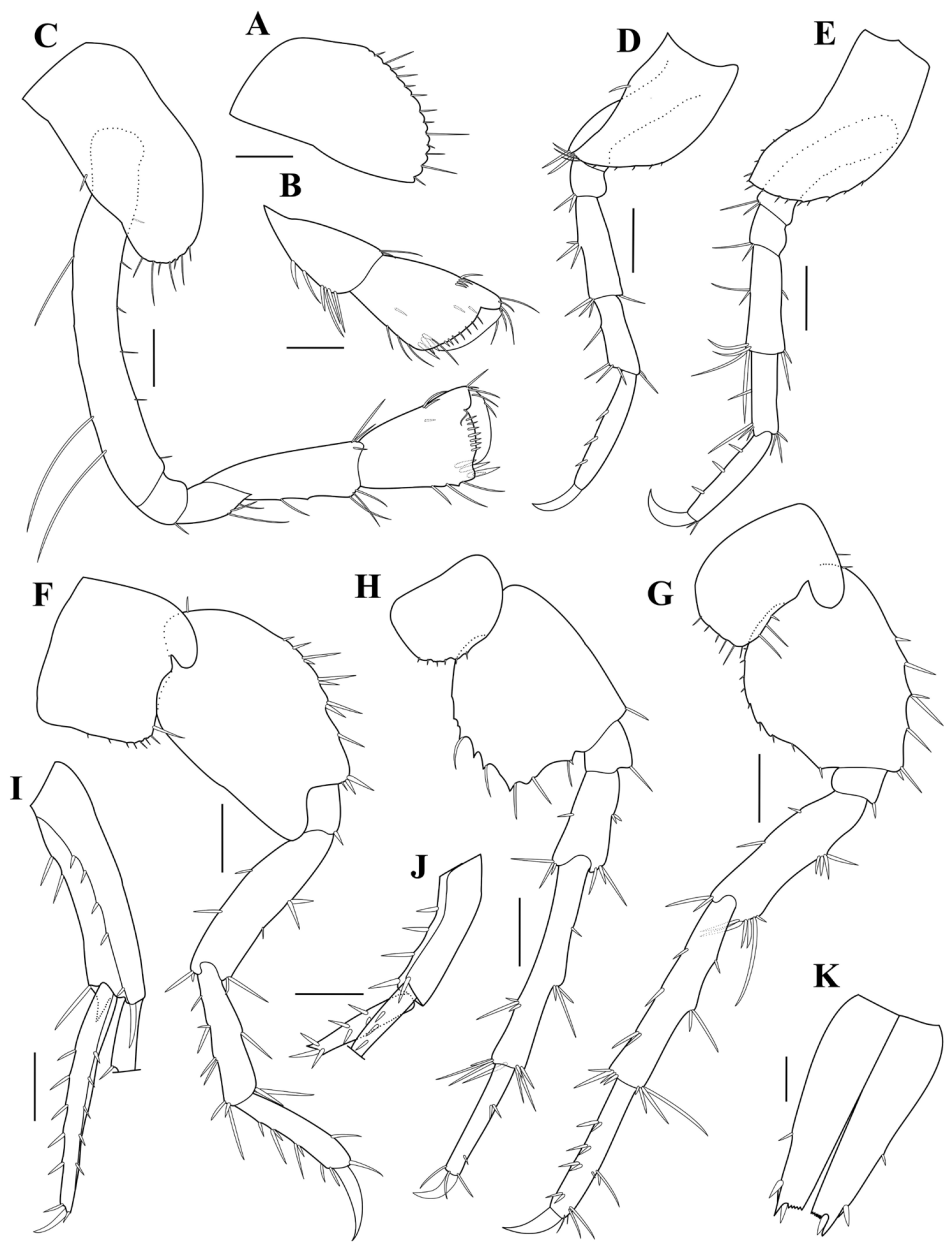
*Paradexaminerotundogena* sp. nov., holotype, adult female, MABIK CR00250814, 5.5 mm **A** coxa 1 **B** gnathopod 1 **C** gnathopod 2 **D** pereopod 3 **E** pereopod 4 **F** pereopod 5 **G** pereopod 6 **H** pereopod 7 **I** uropod 1 **J** uropod 2 **K** telson. Scale bars: 0.1 mm (**A–C**); 0.2 mm (**D–K**).

Figs 2B , 6 , 7 Korean name: Dung-geun-ppyam-yeop-ga-si-but-eun-kko-ri-yeop-sae-u, new

###### Type material.

***Holotype***: female, 5.0 mm, MABIK CR00250814, Korea, Gyeongsangnam-do, Geomundo Island, Guroba, 34°00'52"N, 127°17'41"E, 09 July 2019, scuba collection, depth 10–15 m, S.G. Lee & Y.H. Kim leg. ***Paratypes***: two females, 3.4 mm and 4.5 mm, DKUAMP202203, same station data as holotype.

###### Additional material examined.

1 female, Korea, Gyeongsangnam-do, Geomundo Island, 34°00'43"N, 127°18'05"E, 06 June 2018, S.H. Kim leg. 4 females, Korea, Chujado Island, 33°56'43"N, 126°18'42"E, Z. Xin, K.W. Kim, & Y.H. Kim leg., 28 August 2021.

###### Diagnosis.

Lateral cephalic lobe rounded. Eye medium-sized. Dorsal pleonites tooth formulae 3-3-3-3, rear to front. Antenna 1, peduncular article 2 slightly longer than article 1. Maxilla 1, inner plate without apical seta. Maxilliped, inner plate without lateral setae. Gnathopod 1, propodus broad, palm steeply angled. Pereopods 3–7 spinose. Pereopod 7, basis subquadrate, with irregular serrations posteriorly. Telson deeply cleft nearly to the base.

###### Description.

***Holotype***, **female**, MABIK CR00250814. Body (Fig. 6A) length about 5.0 mm. Cephalic lobe rounded. Eye medium-sized, subround. Pereonites smooth.

Pleonites 1–3 (Figs 6B), dorsal tooth formulae 3-3-3-3, rear to front; pleonal epimera 1‒3 each with posteroventral tooth, its gradually getting bigger distally and posterior margins with irregularly crenulated; urosomite 1 with one dorsodistal carinate tooth and a pair of dorsolateral teeth with a spine.

Antenna 1 (Fig. 6C) slightly longer than half as long as body length; peduncular articles rectangular, length ratio of peduncular articles 1‒3 = 1.00: 1.19: 0.41; accessory flagellum small, with three apical setules; flagellum about 1.3 times as long as peduncle, 15-articulate.

Antenna 2 (Fig. 6D) one-third of the body length and two-thirds of antenna 1 length. peduncular articles 1‒3 short, peduncular articles 4 and 5 elongated, length ratio of peduncular articles 3‒5 = 1.00: 4.14: 4.42; flagellum 7-articulate, 1.4 times as long as peduncular article 5.

Lower lip (Fig. 6E) inner lobe elongate-ovate, covered with patch of pubescence; outer lobe with three or four cusps; mandibular process produced subacutely.

Left mandible (Fig. 6F) incisor produced forward, with eight blunt teeth; lacinia mobilis bifid, upper part with six teeth, lower part with two blunt teeth; three accessory spines placed between lacinia mobilis and molar process; molar process developed, truncate.

Right mandible (Fig. 6G) similar to left mandible, except two accessory spines placed between lacinia mobilis and molar process.

Maxilla 1 (Fig. 6H) inner plate small, elongate, without apical seta; outer plate with 11 tooth-like spines (simple, bifid, and denticulate) apically; palp slender, not reaching end of outer plate, with a long apical seta.

Maxilla 2 (Fig. 6I) inner plate much shorter than outer one, with four apical setae; outer plate with four subapical and three apical setae.

Maxilliped (Fig. 6J) inner plate small, with one apical setae and without lateral setae; outer plate large, elongate-ovate, slightly extending beyond end of palp article 3, inner margin with 15 conical teeth and five simple setae apically; palp 4 articulate, rather slender, inner margin setaceous, slightly extending outer plate.

Gnathopod 1, coxa (Fig. 7A) trapezoidal, anterior margin rounded, with 13 unequal simple setae, posterior margin straight, unarmed; carpus (Fig. 7B) subtriangular, subequal to propodus, with six ventral setae; propodus broad, gradually widening distally, without oblique row of setae medially, palm steeply angled, with a row of short setae, delimited by a group of four spines; dactylus falcate, fitting palm.

Gnathopod 2 (Fig. 7C) similar to gnathopod 1, but coxa narrowly rectangular and carpus elongate, 1.36 times as long as propodus.

Pereopod 3 (Fig. 7D) slender, spinose; coxa tapering distally; length ratio of articles 2‒7 = 1.00: 0.27: 0.70: 0.53: 0.87: 0.40.

Pereopod 4 (Fig. 7E) similar to pereopod 3, except coxa 4 wider than coxa 3 ventrally; length ratio of articles 2‒7 = 1.00: 0.25: 0.69: 0.53: 0.78: 0.38.

Pereopod 5 (Fig. 7F), coxa quadrate, bilobate, anterior rounded lobe protruding downward; basis longish ovate form, posteroventral lobe roundly downward, reaching somewhat distal margin of ischium, with several clusters of long to short spines along anterior margin, posterior margin straight, unarmed; ischium to dactylus slender, setose; length ratio of articles 2‒7 = 1.00: 0.18: 0.70: 0.58: 0.50: 0.34.

Pereopod 6 (Fig. 7G) coxa 6 bilobate, similar to coxa 5, but shallower than coxa 5; basis ovate, posterior margin rounded, weakly serrate, slightly excavate posterodistally; length ratio of articles 2‒7 = 1.00: 0.15: 0.85: 1.08: 0.77: 0.30.

Pereopod 7 (Fig. 7H) coxa small, semicircular; basis subquadrate, width 0.91 times length, produced posteriorly, anterior margin straight, posterior margin with irregular serrations; length ratio of articles 2‒7 = 1.00: 0.26: 0.60: 1.29: 0.72: 0.29.

Uropod 1 (Fig. 7I) peduncle subrectangular, with five dorsolateral, three medial, and one apicolateral large spines; inner ramus slightly shorter than peduncle, with two longitudinal rows of 10 and three apical spines; outer ramus broken.

Uropod 2 (Fig. 7J) about half length of uropod 1, with four dorsolateral and one apicomedial spines; outer ramus 0.75 times as long as peduncle, with two rows of four and three apical spines; inner ramus broken.

Uropod 3 unknown.

Telson (Fig. 7K) longish, 2.00 times as long as wide, thoroughly cleft, lateral margin with two spines, apical margin truncate with acute cusp, serrulation, and one spine.

###### Etymology.

The species name is derived from the Latin *rotundus* (= round) and *gena* (= cheek), referring to the rounded cephalic lobe.

###### Remarks.

The new species *Paradexaminerotundogena* sp. nov. resembles *P.tafunsaka* Myers, 1995 distributed in Kosrae, Micronesia, *P.levitelson* Myers & LeCroy, 2009 from Queensland, Australia, and *P.bisetigera* Hirayama, 1984 from Tomioka Bay, Japan, in having a rounded ocular lobe and dorsal pleonite tooth formulae of 3-3-3-3, rear to front (Table 2). However, the new species is distinguished from its congeners in the following characteristics (compared with the characteristics of congeners in parentheses): 1) gnathopod 2 with carpus longer than propodus (vs subequal in length in *P.bisetigera*, shorter than in *P.tafunsaka* and *P.levitelson*); 2) pereopod 7 with basis broad, with irregular serrations posteriorly (vs regular serrations posteriorly in *P.bisetigera*, *P.levitelson*, and *P.tafunsaka*); 3) maxilla 1 with palp having one apical seta (vs two apical setae in *P.tafunsaka* and *P.bisetigera*, six apical setae in *P.levitelson*); 4) maxilla 2 with inner plate without medial seta (vs with three setae in *P.bisetigera*); 5) telson with two lateral spines (vs five or six lateral spines in *P.bisetigera*, three or four lateral spines in *P.tafunsaka*).

**Table 2.** Morphological characters of *Paradexaminerotundogena* sp. nov. and related species.

Species	*P.tafunsaka* (♀)	*P.bisetigera* (♂)	*P.levitelson* (♀)	*P.rotundogena* sp. nov. (♀)
Characters				
Body length	2.7 mm	6.3 mm	3.0 mm	5.0 mm
Pleonites tooth formulae	3-3-3-3-0	3-3-3-3	3-3-3-3-0	3-3-3-3-0
Ocular lobe	round	round	round	round
Lower lip, corn	2 cones	2 cones	?	3 or 4 cones
Mandibular process	subacutely produced	subacutely produced	?	subacutely produced
Mandible, accessory spines	2 spines	2 or 3 spines	2 spines	2 or 3 spines
Maxilla 1, palp	2 apical setae	2 apical setae	6 apical setae	1 apical seta
Maxilla 2, inner margin of inner plate	?	3 setae	?	no seta
Maxilliped, inner plate	no lateral seta	no lateral seta	?	no lateral seta
Maxilliped, outer plate	12 conical teeth	21 conical teeth	?	15 conical teeth
Antenna 1, articles 1 & 2 ratio	1.0 : 1.3	1.0 : 1.5	1.0 : 1.5	1.0 : 1.2
Gnathopod 1, interior setae of propodus	5 or 6	6	?	3
Gnathopod 1, palm	steeply angled	steeply angled	transverse	steeply angled
Gnathopod 2, carpus & propodus ratio	1.0 : 1.1	1.0 : 1.3	1.0 : 1.3	1.0 : 0.7
Coxa 7	?	semicircular	semicircular	semicircular
Pereopod 7, basis	broad, regular serrations posteriorly	broad, no serrations posteriorly	broad, regular serrations posteriorly	broad, irregular serrations posteriorly
Telson	3 or 4 lateral spines	5 or 6 lateral spines	2 or 3 lateral spines	2 lateral spines
Distribution	Kosrae, Micronesia	Tomioka bay, Japan	Queensland, Australia	Korea
References	Myers, 1995	Hirayama, 1984	Myers & LeCroy, 2009	Present study

###### Distribution.

South Korea (Chujado Island, Geomundo Island).

### ﻿Key to Korean species of *Paradexamine*

**Table:** 

1	Ocular lobe rounded	***P.rotundogena* sp. nov.**
–	Ocular lobe acute	**2**
2	Dorsal pleonite tooth formulate 1-3-3-0	***P.fraudatrix* Tzvetkova, 1976**
–	Dorsal pleonite tooth formulate 1-3-3-3	**3**
3	Antenna 1, peduncular article 2 1.3× article 1; coxa 7 pointed posteroventrally	***P.acuta* sp. nov.**
–	Antenna 1, peduncular article 2 1.5× article 1; coxa 7 rounded posteroventrally	**4**
4	Pereopod 7, basis subrectangular and narrow; telson with a row of lateral spines	***P.jindoensis* Kim & Lee, 2008**
–	Pereopod 7, basis ovate and broad; telson with one lateral spine	***P.gigas* Hirayama, 1984**

## Supplementary Material

XML Treatment for Paradexamine

XML Treatment for Paradexamine acuta

XML Treatment for Paradexamine rotundogena
